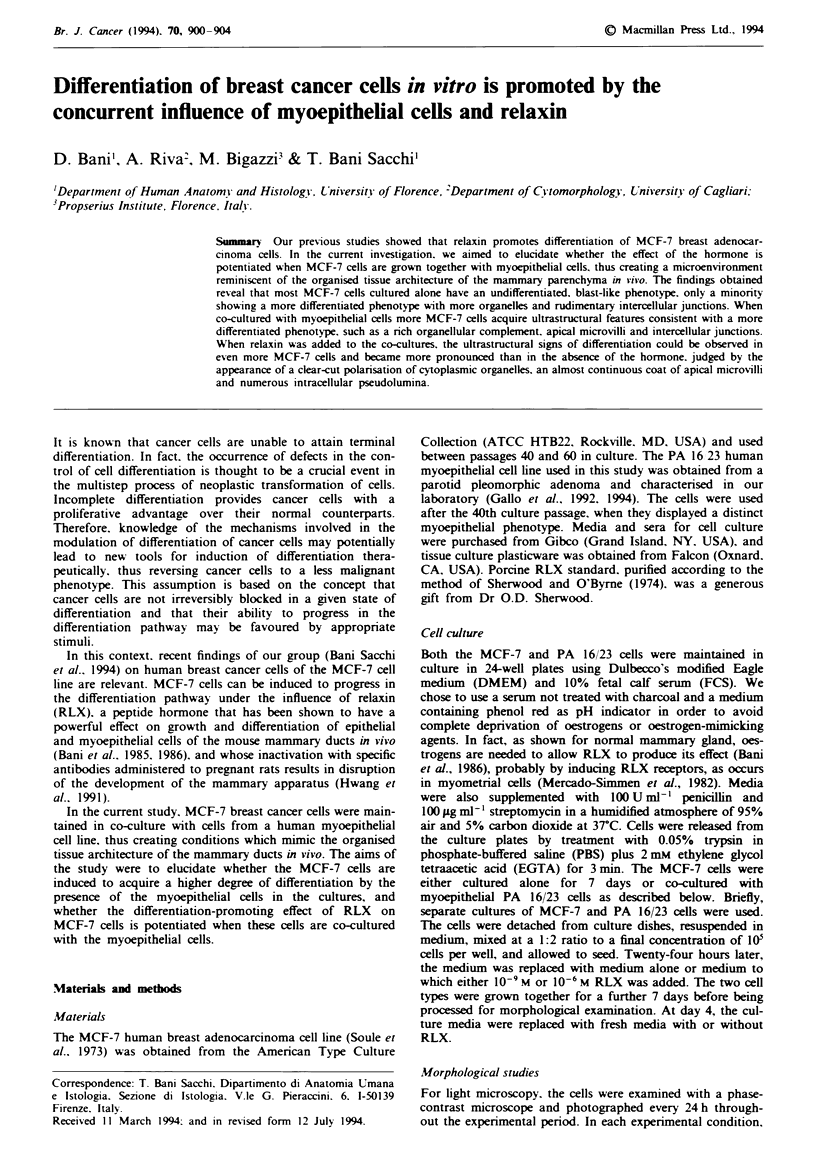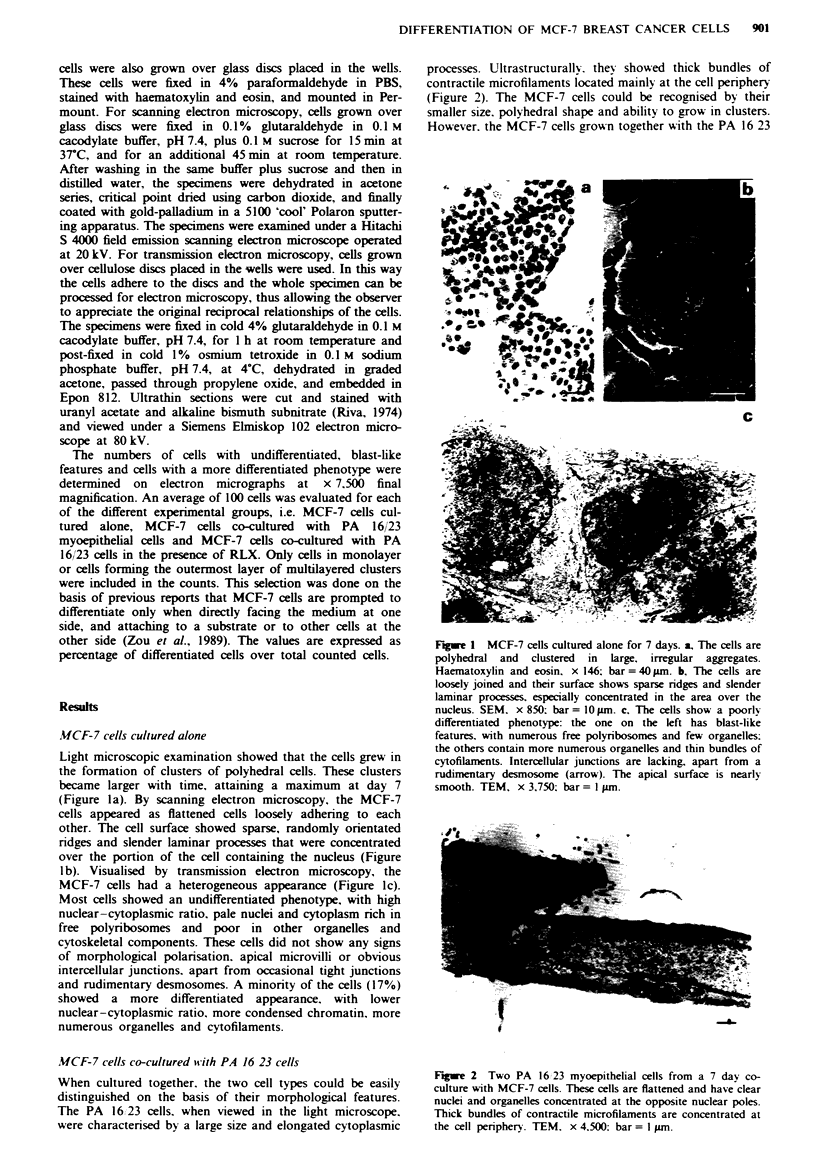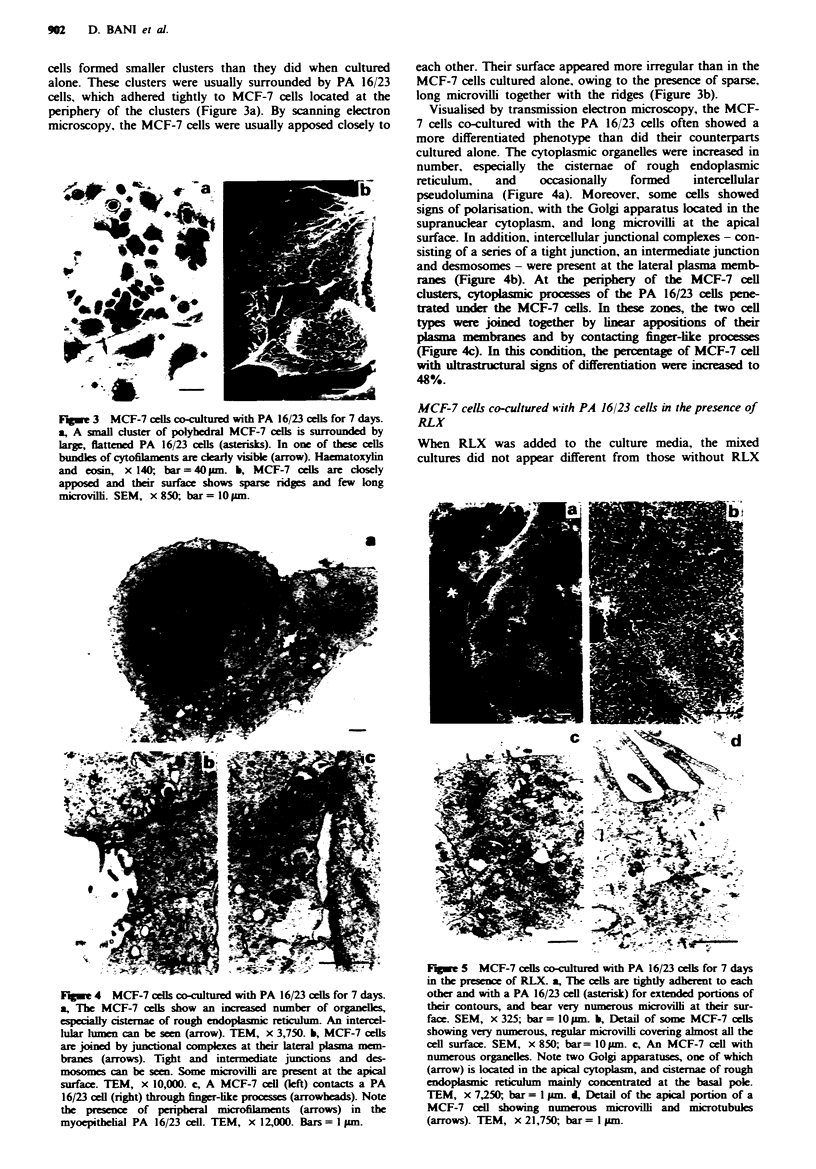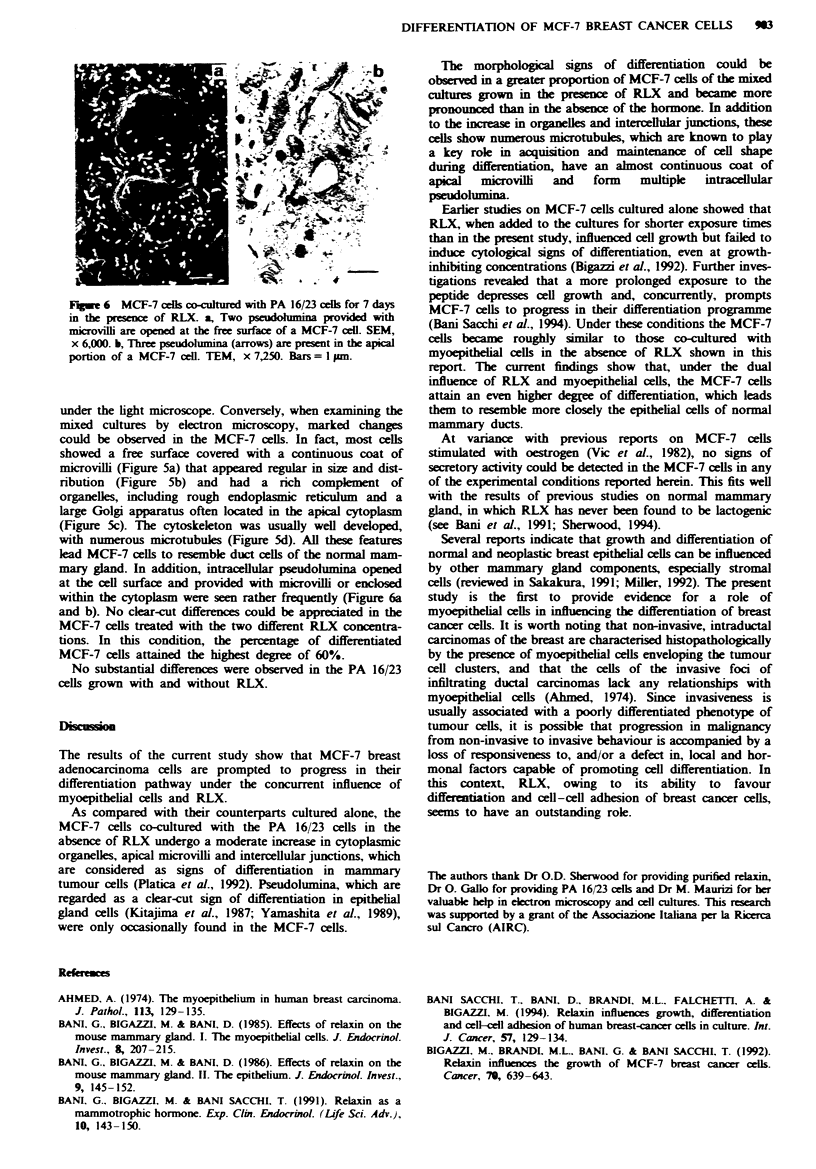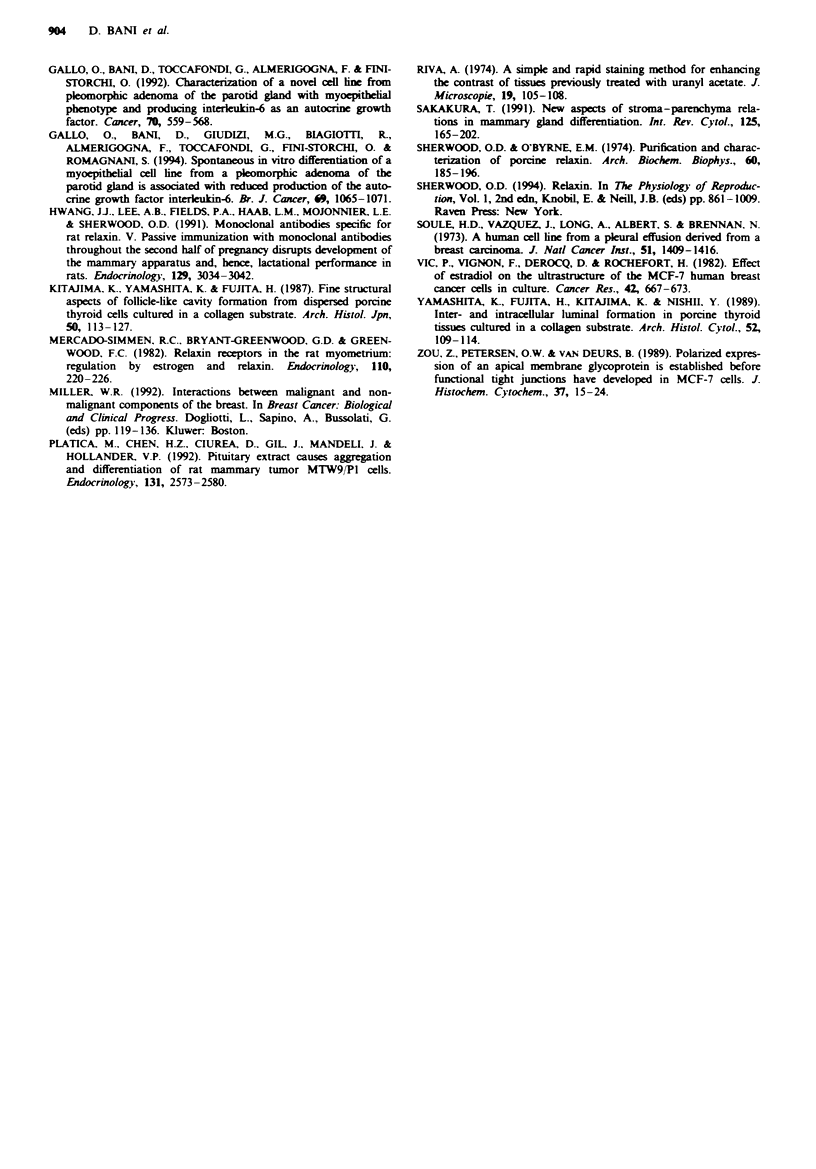# Differentiation of breast cancer cells in vitro is promoted by the concurrent influence of myoepithelial cells and relaxin.

**DOI:** 10.1038/bjc.1994.417

**Published:** 1994-11

**Authors:** D. Bani, A. Riva, M. Bigazzi, T. Bani Sacchi

**Affiliations:** Department of Human Anatomy and Histology, University of Florence, Italy.

## Abstract

**Images:**


Br. J. Cancer (1994), 70, 900-904 © Macmillan Press Ltd., 1994

### Differentiation of breast cancer cells *in vitro* is promoted by the concurrent influence of myoepithelial cells and relaxin

D. Bani[1], A. Riva[2], M. Bigazzi[3] & T. Bani Sacchi[1]

[1]*Department of Human Anatomy and Histology, University of Florence, [2]Department of Cytomorphology, University of Cagliari; [3]Propserius Institute, Florence, Italy.*

**Summary** Our previous studies showed that relaxin promotes differentiation of MCF-7 breast adenocarcinoma cells. In the current investigation, we aimed to elucidate whether the effect of the hormone is potentiated when MCF-7 cells are grown together with myoepithelial cells, thus creating a microenvironment reminiscent of the organised tissue architecture of the mammary parenchyma *in vivo*. The findings obtained reveal that most MCF-7 cells cultured alone have an undifferentiated, blast-like phenotype, only a minority showing a more differentiated phenotype with more organelles and rudimentary intercellular junctions. When co-cultured with myoepithelial cells more MCF-7 cells acquire ultrastructural features consistent with a more differentiated phenotype, such as a rich organellular complement, apical microvilli and intercellular junctions. When relaxin was added to the co-cultures, the ultrastructural signs of differentiation could be observed in even more MCF-7 cells and became more pronounced than in the absence of the hormone, judged by the appearance of a clear-cut polarisation of cytoplasmic organelles, an almost continuous coat of apical microvilli and numerous intracellular pseudolumina.

It is known that cancer cells are unable to attain terminal differentiation. In fact, the occurrence of defects in the control of cell differentiation is thought to be a crucial event in the multistep process of neoplastic transformation of cells. Incomplete differentiation provides cancer cells with a proliferative advantage over their normal counterparts. Therefore, knowledge of the mechanisms involved in the modulation of differentiation of cancer cells may potentially lead to new tools for induction of differentiation therapeutically, thus reversing cancer cells to a less malignant phenotype. This assumption is based on the concept that cancer cells are not irreversibly blocked in a given state of differentiation and that their ability to progress in the differentiation pathway may be favoured by appropriate stimuli.

In this context, recent findings of our group (Bani Sacchi *et al*., 1994) on human breast cancer cells of the MCF-7 cell line are relevant. MCF-7 cells can be induced to progress in the differentiation pathway under the influence of relaxin (RLX), a peptide hormone that has been shown to have a powerful effect on growth and differentiation of epithelial and myoepithelial cells of the mouse mammary ducts *in vivo* (Bani *et al*., 1985, 1986), and whose inactivation with specific antibodies administered to pregnant rats results in disruption of the development of the mammary apparatus (Hwang *et al*., 1991).

In the current study, MCF-7 breast cancer cells were maintained in co-culture with cells from a human myoepithelial cell line, thus creating conditions which mimic the organised tissue architecture of the mammary ducts *in vivo*. The aims of the study were to elucidate whether the MCF-7 cells are induced to acquire a higher degree of differentiation by the presence of the myoepithelial cells in the cultures, and whether the differentiation-promoting effect of RLX on MCF-7 cells is potentiated when these cells are co-cultured with the myoepithelial cells.

### Materials and methods

### Materials

The MCF-7 human breast adenocarcinoma cell line (Soule *et al*., 1973) was obtained from the American Type Culture Collection (ATCC HTB22, Rockville, MD, USA) and used between passages 40 and 60 in culture. The PA 16/23 human myoepithelial cell line used in this study was obtained from a parotid pleomorphic adenoma and characterised in our laboratory (Gallo *et al*., 1992, 1994). The cells were used after the 40th culture passage, when they displayed a distinct myoepithelial phenotype. Media and sera for cell culture were purchased from Gibco (Grand Island, NY, USA), and tissue culture plasticware was obtained from Falcon (Oxnard, CA, USA). Porcine RLX standard, purified according to the method of Sherwood and O'Byrne (1974), was a generous gift from Dr O.D. Sherwood.

### Cell culture

Both the MCF-7 and PA 16/23 cells were maintained in culture in 24-well plates using Dulbecco's modified Eagle medium (DMEM) and 10% fetal calf serum (FCS). We chose to use a serum not treated with charcoal and a medium containing phenol red as pH indicator in order to avoid complete deprivation of oestrogens or oestrogen-mimicking agents. In fact, as shown for normal mammary gland, oestrogens are needed to allow RLX to produce its effect (Bani *et al*., 1986), probably by inducing RLX receptors, as occurs in myometrial cells (Mercado-Simmen *et al*., 1982). Media were also supplemented with $100\ U\ ml^{-1}$ penicillin and $100\ \mu g\ ml^{-1}$ streptomycin in a humidified atmosphere of 95% air and 5% carbon dioxide at 37°C. Cells were released from the culture plates by treatment with 0.05% trypsin in phosphate-buffered saline (PBS) plus 2 mM ethylene glycol tetraacetic acid (EGTA) for 3 min. The MCF-7 cells were either cultured alone for 7 days or co-cultured with myoepithelial PA 16/23 cells as described below. Briefly, separate cultures of MCF-7 and PA 16/23 cells were used. The cells were detached from culture dishes, resuspended in medium, mixed at a 1:2 ratio to a final concentration of $10^5$ cells per well, and allowed to seed. Twenty-four hours later, the medium was replaced with medium alone or medium to which either $10^{-9}$ M or $10^{-6}$ M RLX was added. The two cell types were grown together for a further 7 days before being processed for morphological examination. At day 4, the culture media were replaced with fresh media with or without RLX.

### Morphological studies

For light microscopy, the cells were examined with a phase-contrast microscope and photographed every 24 h throughout the experimental period. In each experimental condition,

Correspondence: T. Bani Sacchi, Dipartimento di Anatomia Umana e Istologia, Sezione di Istologia, V.le G. Pieraccini, 6, I-50139 Firenze, Italy.
Received 11 March 1994; and in revised form 12 July 1994.

cells were also grown over glass discs placed in the wells. These cells were fixed in 4% paraformaldehyde in PBS, stained with haematoxylin and eosin, and mounted in Permount. For scanning electron microscopy, cells grown over glass discs were fixed in 0.1% glutaraldehyde in 0.1 M cacodylate buffer, pH 7.4, plus 0.1 M sucrose for 15 min at 37°C, and for an additional 45 min at room temperature. After washing in the same buffer plus sucrose and then in distilled water, the specimens were dehydrated in acetone series, critical point dried using carbon dioxide, and finally coated with gold-palladium in a 5100 'cool' Polaron sputtering apparatus. The specimens were examined under a Hitachi S 4000 field emission scanning electron microscope operated at 20 kV. For transmission electron microscopy, cells grown over cellulose discs placed in the wells were used. In this way the cells adhere to the discs and the whole specimen can be processed for electron microscopy, thus allowing the observer to appreciate the original reciprocal relationships of the cells. The specimens were fixed in cold 4% glutaraldehyde in 0.1 M cacodylate buffer, pH 7.4, for 1 h at room temperature and post-fixed in cold 1% osmium tetroxide in 0.1 M sodium phosphate buffer, pH 7.4, at 4°C, dehydrated in graded acetone, passed through propylene oxide, and embedded in Epon 812. Ultrathin sections were cut and stained with uranyl acetate and alkaline bismuth subnitrate (Riva, 1974) and viewed under a Siemens Elmiskop 102 electron microscope at 80 kV.

The numbers of cells with undifferentiated, blast-like features and cells with a more differentiated phenotype were determined on electron micrographs at × 7,500 final magnification. An average of 100 cells was evaluated for each of the different experimental groups, i.e. MCF-7 cells cultured alone, MCF-7 cells co-cultured with PA 16/23 myoepithelial cells and MCF-7 cells co-cultured with PA 16/23 cells in the presence of RLX. Only cells in monolayer or cells forming the outermost layer of multilayered clusters were included in the counts. This selection was done on the basis of previous reports that MCF-7 cells are prompted to differentiate only when directly facing the medium at one side, and attaching to a substrate or to other cells at the other side (Zou *et al.*, 1989). The values are expressed as percentage of differentiated cells over total counted cells.

### Results

### *MCF-7 cells cultured alone*

Light microscopic examination showed that the cells grew in the formation of clusters of polyhedral cells. These clusters became larger with time, attaining a maximum at day 7 (Figure 1a). By scanning electron microscopy, the MCF-7 cells appeared as flattened cells loosely adhering to each other. The cell surface showed sparse, randomly orientated ridges and slender laminar processes that were concentrated over the portion of the cell containing the nucleus (Figure 1b). Visualised by transmission electron microscopy, the MCF-7 cells had a heterogeneous appearance (Figure 1c). Most cells showed an undifferentiated phenotype, with high nuclear–cytoplasmic ratio, pale nuclei and cytoplasm rich in free polyribosomes and poor in other organelles and cytoskeletal components. These cells did not show any signs of morphological polarisation, apical microvilli or obvious intercellular junctions, apart from occasional tight junctions and rudimentary desmosomes. A minority of the cells (17%) showed a more differentiated appearance, with lower nuclear–cytoplasmic ratio, more condensed chromatin, more numerous organelles and cytofilaments.

### *MCF-7 cells co-cultured with PA 16/23 cells*

When cultured together, the two cell types could be easily distinguished on the basis of their morphological features. The PA 16/23 cells, when viewed in the light microscope, were characterised by a large size and elongated cytoplasmic processes. Ultrastructurally, they showed thick bundles of contractile microfilaments located mainly at the cell periphery (Figure 2). The MCF-7 cells could be recognised by their smaller size, polyhedral shape and ability to grow in clusters. However, the MCF-7 cells grown together with the PA 16/23

**Figure 1** MCF-7 cells cultured alone for 7 days. **a**, The cells are polyhedral and clustered in large, irregular aggregates. Haematoxylin and eosin, × 146; bar = 40 μm. **b**, The cells are loosely joined and their surface shows sparse ridges and slender laminar processes, especially concentrated in the area over the nucleus. SEM, × 850; bar = 10 μm. **c**, The cells show a poorly differentiated phenotype: the one on the left has blast-like features, with numerous free polyribosomes and few organelles; the others contain more numerous organelles and thin bundles of cytofilaments. Intercellular junctions are lacking, apart from a rudimentary desmosome (arrow). The apical surface is nearly smooth. TEM, × 3,750; bar = 1 μm.

**Figure 2** Two PA 16/23 myoepithelial cells from a 7 day co-culture with MCF-7 cells. These cells are flattened and have clear nuclei and organelles concentrated at the opposite nuclear poles. Thick bundles of contractile microfilaments are concentrated at the cell periphery. TEM, × 4,500; bar = 1 μm.

cells formed smaller clusters than they did when cultured alone. These clusters were usually surrounded by PA 16/23 cells, which adhered tightly to MCF-7 cells located at the periphery of the clusters (Figure 3a). By scanning electron microscopy, the MCF-7 cells were usually apposed closely to each other. Their surface appeared more irregular than in the MCF-7 cells cultured alone, owing to the presence of sparse, long microvilli together with the ridges (Figure 3b).

Visualised by transmission electron microscopy, the MCF-7 cells co-cultured with the PA 16/23 cells often showed a more differentiated phenotype than did their counterparts cultured alone. The cytoplasmic organelles were increased in number, especially the cisternae of rough endoplasmic reticulum, and occasionally formed intercellular pseudolumina (Figure 4a). Moreover, some cells showed signs of polarisation, with the Golgi apparatus located in the supranuclear cytoplasm, and long microvilli at the apical surface. In addition, intercellular junctional complexes – consisting of a series of a tight junction, an intermediate junction and desmosomes – were present at the lateral plasma membranes (Figure 4b). At the periphery of the MCF-7 cell clusters, cytoplasmic processes of the PA 16/23 cells penetrated under the MCF-7 cells. In these zones, the two cell types were joined together by linear appositions of their plasma membranes and by contacting finger-like processes (Figure 4c). In this condition, the percentage of MCF-7 cell with ultrastructural signs of differentiation were increased to 48%.

**Figure 3** MCF-7 cells co-cultured with PA 16/23 cells for 7 days. **a**, A small cluster of polyhedral MCF-7 cells is surrounded by large, flattened PA 16/23 cells (asterisks). In one of these cells bundles of cytofilaments are clearly visible (arrow). Haematoxylin and eosin, × 140; bar = 40 µm. **b**, MCF-7 cells are closely apposed and their surface shows sparse ridges and few long microvilli. SEM, × 850; bar = 10 µm.

**Figure 4** MCF-7 cells co-cultured with PA 16/23 cells for 7 days. **a**, The MCF-7 cells show an increased number of organelles, especially cisternae of rough endoplasmic reticulum. An intercellular lumen can be seen (arrow). TEM, × 3,750. **b**, MCF-7 cells are joined by junctional complexes at their lateral plasma membranes (arrows). Tight and intermediate junctions and desmosomes can be seen. Some microvilli are present at the apical surface. TEM, × 10,000. **c**, A MCF-7 cell (left) contacts a PA 16/23 cell (right) through finger-like processes (arrowheads). Note the presence of peripheral microfilaments (arrows) in the myoepithelial PA 16/23 cell. TEM, × 12,000. Bars = 1 µm.

*MCF-7 cells co-cultured with PA 16/23 cells in the presence of RLX*

When RLX was added to the culture media, the mixed cultures did not appear different from those without RLX

**Figure 5** MCF-7 cells co-cultured with PA 16/23 cells for 7 days in the presence of RLX. **a**, The cells are tightly adherent to each other and with a PA 16/23 cell (asterisk) for extended portions of their contours, and bear very numerous microvilli at their surface. SEM, × 325; bar = 10 µm. **b**, Detail of some MCF-7 cells showing very numerous, regular microvilli covering almost all the cell surface. SEM, × 850; bar = 10 µm. **c**, An MCF-7 cell with numerous organelles. Note two Golgi apparatuses, one of which (arrow) is located in the apical cytoplasm, and cisternae of rough endoplasmic reticulum mainly concentrated at the basal pole. TEM, × 7,250; bar = 1 µm. **d**, Detail of the apical portion of a MCF-7 cell showing numerous microvilli and microtubules (arrows). TEM, × 21,750; bar = 1 µm.

**Figure 6** MCF-7 cells co-cultured with PA 16/23 cells for 7 days in the presence of RLX. **a**, Two pseudolumina provided with microvilli are opened at the free surface of a MCF-7 cell. SEM, × 6,000. **b**, Three pseudolumina (arrows) are present in the apical portion of a MCF-7 cell. TEM, × 7,250. Bars = 1 μm.

under the light microscope. Conversely, when examining the mixed cultures by electron microscopy, marked changes could be observed in the MCF-7 cells. In fact, most cells showed a free surface covered with a continuous coat of microvilli (Figure 5a) that appeared regular in size and distribution (Figure 5b) and had a rich complement of organelles, including rough endoplasmic reticulum and a large Golgi apparatus often located in the apical cytoplasm (Figure 5c). The cytoskeleton was usually well developed, with numerous microtubules (Figure 5d). All these features lead MCF-7 cells to resemble duct cells of the normal mammary gland. In addition, intracellular pseudolumina opened at the cell surface and provided with microvilli or enclosed within the cytoplasm were seen rather frequently (Figure 6a and b). No clear-cut differences could be appreciated in the MCF-7 cells treated with the two different RLX concentrations. In this condition, the percentage of differentiated MCF-7 cells attained the highest degree of 60%.

No substantial differences were observed in the PA 16/23 cells grown with and without RLX.

### Discussion

The results of the current study show that MCF-7 breast adenocarcinoma cells are prompted to progress in their differentiation pathway under the concurrent influence of myoepithelial cells and RLX.

As compared with their counterparts cultured alone, the MCF-7 cells co-cultured with the PA 16/23 cells in the absence of RLX undergo a moderate increase in cytoplasmic organelles, apical microvilli and intercellular junctions, which are considered as signs of differentiation in mammary tumour cells (Platica *et al.*, 1992). Pseudolumina, which are regarded as a clear-cut sign of differentiation in epithelial gland cells (Kitajima *et al.*, 1987; Yamashita *et al.*, 1989), were only occasionally found in the MCF-7 cells.

The morphological signs of differentiation could be observed in a greater proportion of MCF-7 cells of the mixed cultures grown in the presence of RLX and became more pronounced than in the absence of the hormone. In addition to the increase in organelles and intercellular junctions, these cells show numerous microtubules, which are known to play a key role in acquisition and maintenance of cell shape during differentiation, have an almost continuous coat of apical microvilli and form multiple intracellular pseudolumina.

Earlier studies on MCF-7 cells cultured alone showed that RLX, when added to the cultures for shorter exposure times than in the present study, influenced cell growth but failed to induce cytological signs of differentiation, even at growth-inhibiting concentrations (Bigazzi *et al.*, 1992). Further investigations revealed that a more prolonged exposure to the peptide depresses cell growth and, concurrently, prompts MCF-7 cells to progress in their differentiation programme (Bani Sacchi *et al.*, 1994). Under these conditions the MCF-7 cells became roughly similar to those co-cultured with myoepithelial cells in the absence of RLX shown in this report. The current findings show that, under the dual influence of RLX and myoepithelial cells, the MCF-7 cells attain an even higher degree of differentiation, which leads them to resemble more closely the epithelial cells of normal mammary ducts.

At variance with previous reports on MCF-7 cells stimulated with oestrogen (Vic *et al.*, 1982), no signs of secretory activity could be detected in the MCF-7 cells in any of the experimental conditions reported herein. This fits well with the results of previous studies on normal mammary gland, in which RLX has never been found to be lactogenic (see Bani *et al.*, 1991; Sherwood, 1994).

Several reports indicate that growth and differentiation of normal and neoplastic breast epithelial cells can be influenced by other mammary gland components, especially stromal cells (reviewed in Sakakura, 1991; Miller, 1992). The present study is the first to provide evidence for a role of myoepithelial cells in influencing the differentiation of breast cancer cells. It is worth noting that non-invasive, intraductal carcinomas of the breast are characterised histopathologically by the presence of myoepithelial cells enveloping the tumour cell clusters, and that the cells of the invasive foci of infiltrating ductal carcinomas lack any relationships with myoepithelial cells (Ahmed, 1974). Since invasiveness is usually associated with a poorly differentiated phenotype of tumour cells, it is possible that progression in malignancy from non-invasive to invasive behaviour is accompanied by a loss of responsiveness to, and/or a defect in, local and hormonal factors capable of promoting cell differentiation. In this context, RLX, owing to its ability to favour differentiation and cell–cell adhesion of breast cancer cells, seems to have an outstanding role.

The authors thank Dr O.D. Sherwood for providing purified relaxin, Dr O. Gallo for providing PA 16/23 cells and Dr M. Maurizi for her valuable help in electron microscopy and cell cultures. This research was supported by a grant of the Associazione Italiana per la Ricerca sul Cancro (AIRC).

### References

AHMED, A. (1974). The myoepithelium in human breast carcinoma. *J. Pathol.*, **113**, 129–135.

BANI, G., BIGAZZI, M. & BANI, D. (1985). Effects of relaxin on the mouse mammary gland. I. The myoepithelial cells. *J. Endocrinol. Invest.*, **8**, 207–215.

BANI, G., BIGAZZI, M. & BANI, D. (1986). Effects of relaxin on the mouse mammary gland. II. The epithelium. *J. Endocrinol. Invest.*, **9**, 145–152.

BANI, G., BIGAZZI, M. & BANI SACCHI, T. (1991). Relaxin as a mammotrophic hormone. *Exp. Clin. Endocrinol. (Life Sci. Adv.)*, **10**, 143–150.

BANI SACCHI, T., BANI, D., BRANDI, M.L., FALCHETTI, A. & BIGAZZI, M. (1994). Relaxin influences growth, differentiation and cell–cell adhesion of human breast-cancer cells in culture. *Int. J. Cancer*, **57**, 129–134.

BIGAZZI, M., BRANDI, M.L., BANI, G. & BANI SACCHI, T. (1992). Relaxin influences the growth of MCF-7 breast cancer cells. *Cancer*, **70**, 639–643.

GALLO, O., BANI, D., TOCCAFONDI, G., ALMERIGOGNA, F. & FINI-STORCHI, O. (1992). Characterization of a novel cell line from pleomorphic adenoma of the parotid gland with myoepithelial phenotype and producing interleukin-6 as an autocrine growth factor. *Cancer*, **70**, 559-568.

GALLO, O., BANI, D., GIUDIZI, M.G., BIAGIOTTI, R., ALMERIGOGNA, F., TOCCAFONDI, G., FINI-STORCHI, O. & ROMAGNANI, S. (1994). Spontaneous in vitro differentiation of a myoepithelial cell line from a pleomorphic adenoma of the parotid gland is associated with reduced production of the autocrine growth factor interleukin-6. *Br. J. Cancer*, **69**, 1065-1071.

HWANG, J.J., LEE, A.B., FIELDS, P.A., HAAB, L.M., MOJONNIER, L.E. & SHERWOOD, O.D. (1991). Monoclonal antibodies specific for rat relaxin. V. Passive immunization with monoclonal antibodies throughout the second half of pregnancy disrupts development of the mammary apparatus and, hence, lactational performance in rats. *Endocrinology*, **129**, 3034-3042.

KITAJIMA, K., YAMASHITA, K. & FUJITA, H. (1987). Fine structural aspects of follicle-like cavity formation from dispersed porcine thyroid cells cultured in a collagen substrate. *Arch. Histol. Jpn*, **50**, 113-127.

MERCADO-SIMMEN, R.C., BRYANT-GREENWOOD, G.D. & GREENWOOD, F.C. (1982). Relaxin receptors in the rat myometrium: regulation by estrogen and relaxin. *Endocrinology*, **110**, 220-226.

MILLER, W.R. (1992). Interactions between malignant and non-malignant components of the breast. In *Breast Cancer: Biological and Clinical Progress*. Dogliotti, L., Sapino, A., Bussolati, G. (eds) pp. 119-136. Kluwer: Boston.

PLATICA, M., CHEN, H.Z., CIUREA, D., GIL, J., MANDELI, J. & HOLLANDER, V.P. (1992). Pituitary extract causes aggregation and differentiation of rat mammary tumor MTW9/P1 cells. *Endocrinology*, **131**, 2573-2580.

RIVA, A. (1974). A simple and rapid staining method for enhancing the contrast of tissues previously treated with uranyl acetate. *J. Microscopie*, **19**, 105-108.

SAKAKURA, T. (1991). New aspects of stroma-parenchyma relations in mammary gland differentiation. *Int. Rev. Cytol.*, **125**, 165-202.

SHERWOOD, O.D. & O'BYRNE, E.M. (1974). Purification and characterization of porcine relaxin. *Arch. Biochem. Biophys.*, **60**, 185-196.

SHERWOOD, O.D. (1994). Relaxin. In *The Physiology of Reproduction*, Vol. 1, 2nd edn, Knobil, E. & Neill, J.B. (eds) pp. 861-1009. Raven Press: New York.

SOULE, H.D., VAZQUEZ, J., LONG, A., ALBERT, S. & BRENNAN, N. (1973). A human cell line from a pleural effusion derived from a breast carcinoma. *J. Natl Cancer Inst.*, **51**, 1409-1416.

VIC, P., VIGNON, F., DEROCQ, D. & ROCHEFORT, H. (1982). Effect of estradiol on the ultrastructure of the MCF-7 human breast cancer cells in culture. *Cancer Res.*, **42**, 667-673.

YAMASHITA, K., FUJITA, H., KITAJIMA, K. & NISHII, Y. (1989). Inter- and intracellular luminal formation in porcine thyroid tissues cultured in a collagen substrate. *Arch. Histol. Cytol.*, **52**, 109-114.

ZOU, Z., PETERSEN, O.W. & VAN DEURS, B. (1989). Polarized expression of an apical membrane glycoprotein is established before functional tight junctions have developed in MCF-7 cells. *J. Histochem. Cytochem.*, **37**, 15-24.